# Transient left ventricular systolic dysfunction following surgical closure of large patent ductus arteriosus among children and adolescents operated at the cardiac centre, Ethiopia

**DOI:** 10.1186/1749-8090-8-139

**Published:** 2013-05-31

**Authors:** Birkneh Tilahun, Endale Tefera

**Affiliations:** 1 Department of Pediatrics and Child Health, Hawassa University, Hawassa, Ethiopia; 2 Departments of Pediatrics and Child Health, Addis Ababa University, Addis Ababa, Ethiopia

**Keywords:** Large PDA closure, Highland, Systolic Dysfunction

## Abstract

**Background:**

Patent ductus arteriosus (PDA) is one of the commonest congenital heart diseases that require closure within the first few months after birth. The residential area of patients affects the size of the PDA: living in highlands, like most places in Ethiopia, is a risk for having larger sized PDA. Closure of these congenital heart defects is usually performed at an early age in places where capable centers are available. In Ethiopia, closure of these defects is done on mission basis often at an older age. Recently, limited reports came about the occurrence of postoperative left ventricular systolic dysfunction (POLVD) following closure of PDA though full explanation is still lacking.

**Objective:**

To determine the rate of and time to improvement of POLVD and the factors associated with it in children and adolescents who underwent surgical closure of PDA.

**Method:**

All children and adolescents who underwent surgical closure of PDA at the Cardiac Center, Ethiopia (CCE) had postoperative follow up with echocardiography. Serial left ventricular ejection fraction (LVEF) and fiber shortening (FS) values were recorded for all of them. SPSS 20 was used to analyze the data.

**Results:**

A total of 36 children and adolescents who underwent surgical closure of PDA from January 2009 to December 2012 and who fulfilled the inclusion criteria were studied. Their mean age at intervention was 8.52 years (SD = 5.23 years), 77.80% were females. The mean duct size as determined by either echocardiography or intra-operative by the surgeon was 10.31 mm (SD = 3.20 mm). They were followed for a mean duration of 24.80 months (SD = 12.36 months) following surgical closure of PDA. The mean LVEF and FS decreased from 65.06% and 35.28% preoperatively to 54.83% and 28.40% post-operatively respectively. Fifteen (42.86%) of the patients had a post-operative LVEF of less than 55%. The mean time to normalization of systolic function was 5.11 weeks (SD = 3.30 weeks). Having an associated cardiac lesion was an independent predictor of POLVD.

**Conclusions:**

We conclude that there is a high rate of POLVD following surgical closure of large PDA in highlanders. We recommend a serial and systematic follow up of these children postoperatively. Those with a significant cardiac dysfunction may need cardiac medications like Angiotensin Converting Enzyme Inhibitors (ACEI).

## Background

PDA is one of the commonest congenital cardiovascular abnormalities and its incidence is much higher (about thirty times greater) at high altitude as compared to sea level [1]. A recent study which compared the anatomic and hemodynamic features of PDA at high and low altitudes by Jacek *et al.* showed a significantly larger PDA diameter for the former (2.3 ± 1.3 mm vs 4.1 ± 1.2 respectively; *P*-value < 0.001) [2]. A large PDA is often symptomatic as compared to the smaller ones. Irrespective of age and size, all PDAs need either surgical or catheter closure. The small ones should be closed to prevent the risk of bacterial endocarditis or other late complications; where as in moderate to large PDAs the main purpose of the closure is preventing heart failure and/or pulmonary vascular disease [3].

Ventricular dysfunction following PDA ligation through surgical or percutaneous methods has been reported by various studies elsewhere [4-8]. In a Saud Arabian study, Omar *et al.* found that closure of large PDA in children was associated with an immediate deterioration of left ventricular performance (FS and LVEF fell from 37.4% and 67.8% pre-closure to 27.7% and 54.5% post-closure, respectively), which appeared to recover within a few months [4].

Most patients in the Ethiopian context are not diagnosed early and are expected to have a larger PDA owing to the high altitude of most residential places in the country. It appears that these patients represent a distinct population living at a high altitude and presenting late for medical care and intervention. There is a scarcity of studies on this group of patients especially how well they fare following a delayed intervention. This article reports on the occurrence of and factors associated with left ventricular dysfunction following surgical closure of PDA.

## Methods

### Setting

The CCE is one of the few cardiac surgery centers in Africa and, indeed it is the only center in Ethiopia where pediatric cardiac surgery and percutaneous intervention of congenital and acquired cardiac problems is performed. The center is a charity establishment inaugurated in Jan 2009. Though diagnostic procedures and percutaneous interventions could be performed whenever consumables are available, open heart surgeries are solely dependent on surgical missions from overseas who come on voluntary basis. Patients who underwent closed or open heart surgeries or percutaneous interventions underwent daily echocardiograms till discharge. Most patients were seen on the third day of discharge. Then, patients who underwent surgical procedures were transferred to the pediatric cardiac follow up clinic of Tikur Anbessa Specialized Hospital. Patients with catheter interventions continue their follow up at the cardiac center. All echocardiograms including diagnosis, preoperative screening and follow up were performed by consultant pediatric cardiologists.

### Echocardiography

Postoperatively, echocardiography was done daily or every two days within the first 2 weeks. The first recorded echocardiographic report was taken as the first postoperative imaging for the current study; hence, it is possible that an earlier imaging was missed. Mean normal value of fractional shortening was taken as 36%, with 95% prediction limits of 28% to 44%; and the normal mean ejection fraction was taken as 66% with a range of 56% to 78% [9].

### Patients

In this cohort, children and adolescents who underwent surgical closure of PDA from January 2009 to December 2012 at the CCE were included. Patients who underwent surgical closure of PDA but had associated lesions requiring open heart surgery (cardiopulmonary bypass) were excluded from the study.

Over three years, a total of 40 cases underwent surgical closure of PDA; these cases represented patients without other associated congenital or acquired cardiac diagnoses that could be responsible for the left ventricular systolic dysfunction postoperatively which is the main theme of this study. Of the 40 cases, two were excluded because of incomplete recording of their postoperative cardiac function. Two more patients with PDA surgery were also excluded because the surgery was under bypass for an embolized device during percutaneous closure.

### Statistical analysis

Data were entered into SPSS 20 for windows; descriptive statistics were employed to summarize variables. Using binary regression, association of various variables with LVEF values was computed. All the variables with a P—value of <0.2 were then entered in to a multivariable logistic model to control confounders. Finally variables with P-value <0.05 after controlling confounders were retained as independent factors. 95% confidence intervals were generated using the logistic models.

Ethical approval was obtained from the Addis Ababa University, College of Medicine and Health Sciences Institutional Review Board (IRB).

## Results

The mean age for the cohort was 8.52 years (SD = 5.23 years) at intervention, and 77.80% were females. There was one intra-operative death due to iatrogenic aortic perforation. They were followed for a mean duration of 24.80 months (SD = 12.36 months) after surgical closure of PDA.

The mean preoperative LVEF of the cohort was 65.06% (SD = 4.12%); and a mean fractional shortening of 35.28% (SD = 3.02%). The mean postoperative LVEF for the whole cohort was 54.83% (SD = 9.77%); while the mean fractional shortening was 28.34% (SD = 6.21%). Of the 35 patients for whom postoperative LVEF was determined, 15 (42.86%) had a reduced LVEF postoperatively. The mean time to normalization of systolic function was determined for 9 of the patients and was 5.11 weeks (SD = 3.30 weeks). Two of the patients with POLVD disappeared from the follow up; and the other 4 patients were included in the study shortly after operation and are still on follow up with POLVD. All of these children required post-operative ACEI and diuretics.

The mean time to the first postoperative echocardiogram was 5.55 days (SD = 2.50 days). During the immediate postoperative period, 9(25%) patients had complications which included: systemic hypertension, 4/36(11.11%); atelectasis, 1/36(2.78%); pleural effusion, 1/36(2.78%); chylothorax, 1/36(2.78%); hematoma, 1/36(2.78%); subcutaneous emphysema, 1/36(2.78%); and pneumothorax, 1/36(2.78%).

**Figure 1:**
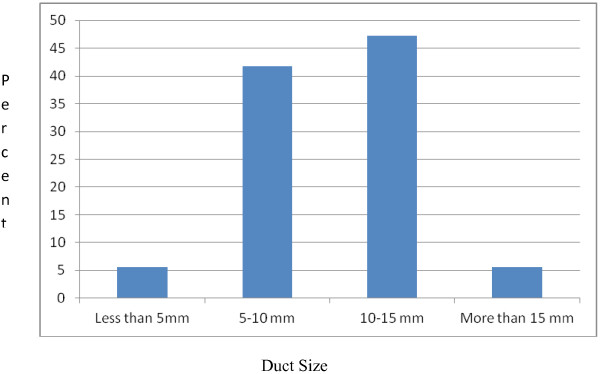
Distribution of the duct size across the cohort of patients who underwent surgical closure at the cardiac center, Ethiopia, 2009–2012.

The mean duct size as determined by either echocardiography or intra-operative by the surgeon was 10.31 mm (SD = 3.20 mm); the smallest and largest duct sizes were 5 mm and 18 mm respectively. (Figure 1) The mean PDA size was larger (10.90 ± 3.46 mm) for children who had postoperative systolic dysfunction as compared to children with a normal systolic function (9.50 ± 3.05 mm); though this was not statistically significant. Most of the patients (80.60%) in the cohort were New York Heart Association (NYHA) class II, only 4(11.11%) and 3(8.30%) were NYHA class III and I respectively.

Fourteen patients (38.90%) had associated lesions/anomaly: minimal to moderate mitral regurgitation, 7(19.44%); coarctaion of the aorta, 5(13.90%); and one had a tiny atrial septal defect (ASD) (which did not need closure) and another one had skeletal anomaly, 1(2.78%). Nine (64.28%) of the patients with associated lesions/anomalies had POLVD.

While testing for association, taking preoperative medications (P-value = 0.04; Crude Odds Ratio (COR), 4.96 95% Confidence Interval (CI), 1.01-24.37), and having associated cardiac lesions (P-value = 0.02; COR, 6.00 95% CI 1.33-27.05) were associated with increased odds of having post operative systolic dysfunction. While both were entered to a multivariable logistic model, the presence of associated cardiac lesions was the only independent predictor of POLVD, (Adjusted odds ratio (AOR), 4.23 95% CI 1.02-21.16) (Table 1).

**Table 1:** Bivariate and multivariable logistic regression models of children who underwent surgical closure of patent ductus arteriosus at Addis Ababa University, Cardiac Centre Ethiopia

	**Postoperative LVEF® (%)**		***P - Value***	**COR (95% CI)**	**AOR(95% CI)**
	**< 55**	**>/cpa55**			
Age at diagnosis					
- Less than 60 mo*	10(28.6)	10(28.6)	0.47	2.00(0.30-13.51)	-
- 60 mo -120 mo	3(8.6)	4(11.4)	0.72	1.50(0.16-14.42)	
- More than 120 mo	2(6.4)	4(11.4)	1	1	
Age at intervention					
- Less than 60 mo	6(17.1)	6(17.1)	0.27	2.67(0.47-15.25)	-
- 60 mo -120 mo	6(17.1)	6(17.1)	0.27	2.67(0.47-15.25)	
- More than 120 mo	3(8.6)	8(22.8)	1	1	
Sex/Gender					
- Male	5(14.3)	3(8.6)	0.21	2.83(0.56-14.47)	-
- Female	10(28.6)	17(48.6)	1	1	
Weight for age					
- < -2ZS^$^	6(17.1)	10(28.6)	0.56	1.50(0.40-5.81)	-
- -2 to +2ZS	5(14.3)	10(28.6)	1	1	
Height for age					
- < -2ZS	9(25.7)	8(22.8)	0.30	2.06(0.52-8.18)	-
- -2 to +2ZS	6(17.1)	11(31.5)	1	1	
Duct size					
- <10 mmß	3(8.6)	8(22.8)	0.21	0.38(0.08-1.78)	-
- ≥10 mm	12(34.3)	12(34.3)	1		
Preop LVED					
- > +3ZS	11(35.5)	16(51.0)	0.64	1.46(0.30-7.09)	-
- < +3ZS	4(11.4)	4(11.4)	1	1	
Preop FS (%)£					
- 35 or less	7(20.0)	8(22.8)	0.69	1.31(0.34-5.08)	-
- More than 35	8(22.8)	12(34.3)	1	1	
PAH^€^					
- Absent	6(17.1)	9(25.7)	0.78	1.22(0.32-4.77)	-
- Present	9(25.7)	11(31.5)	1	1	
Preop¥ cardiac medication					
- Yes	7(20.0)	3(8.6)	0.04	4.96(1.01-24.37)	2.89(0.51-16.64)
- No	8(22.8)	17(48.6)	1	1	1
Associated lesions					
- Yes	9(25.7)	4(11.4)	0.02	6.00(1.33-27.05)	4.23(1.02-21.16)
- No	6(17.1)	16(45.7)	1	1	1
ICU# Stay					
- Less than 2 days	7(22.6)	11(35.5)	0.41	0.55(0.13-2.31)	-
- More than 2 days	6(19.4)	7(22.6)	1	1	
Residual shunt					
- Yes	2(6.4)	2(6.4)	0.76	1.40(0.17-11.14)	-
- No	13(37.1)	18(51.4)	1	1	

Months, $ Z-score, # Intensive Care Unit, € Pulmonary Arterial Hypertension, ¥ Preoperative , £ Preoperative Fiber Shortening Percentage, ß Millimeters, ® Left Ventricular Ejection Fraction.

## Discussion

In the current study, the mean age at intervention was 8.52 years and the PDA size was large for all the patients. There was a reduction in the mean LVEF and FS following surgical closure of the PDAs as compared to the mean preoperative values. The proportion of children with POLVD was 42.86% of the total cohort and the mean time to normalization was 5.11 weeks. Presence of associated cardiac lesions (mitral regurgitation, coarctation of aorta and tiny ASD) was found to be an independent predictor of POLVD after controlling for other variables.

The mean PDA size for this cohort was larger than most other studies elsewhere [2,7,10-13]. This is possibly due to the high altitude of residential areas of most patients in Ethiopia. The relationship between altitude and PDA size was described by other studies [1,2]. The mean age of PDA closure for the current study was older than other studies elsewhere [4,7,13,14]. This is possibly because of delayed presentation of patients to health facility (reflecting the poor healthcare system) and/or lack of surgical facilities prior to the opening of the CCE.

Despite the late intervention and large PDA size in the current cohort, the rate of systolic dysfunction diagnosed by echocardiography was lower than or similar to other studies [12,14]. But, it was higher than one study which found that 25% of patients had post device closure systolic dysfunction at day one postoperatively; the mean age was younger than the current study [7]. The time to improvement of the systolic function observed in the current study was faster than studies by Gupta *et al.*, Galal *et al.* and others [4,5,7]; but longer than one study of preterm infants who underwent ligation within a few hours of delivery who showed improvement in the cardiac systolic function within nearly 24 hours. The authors of the study suggested a decrease in preload as the possible mechanism though it was difficult to explain the fast improvement [13].

Studies elsewhere showed that lower pre-closure FS/LVEF was associated with a higher chance of having postoperative systolic dysfunction; however, in the current study, only the presence of associated cardiac lesions was the independent predictor of POLVD [7,15].

The small sample size of the current study could have hindered the possibility of eliciting small differences in the variables affecting the development of systolic dysfunction. The lack of regular recordings of echocardiographic parameters in the postoperative period may have missed children with systolic dysfunction who had a rapid improvement in their LV systolic function.

## Conclusions

There was a high rate of occurrence of POLVD in patients who have a large sized PDA and in those who underwent the procedure at an older age. All the children with postoperative systolic dysfunction had a normal function in less than 3 months (mean 5 weeks). Presence of associated lesions was the only independent predictor of post-operative LV systolic dysfunction. Based on these findings, we recommend routine follow up of patients who underwent PDA ligation with echocardiography and possible initiation of ACEI for those with significant LV systolic dysfunction.

## Competing interest

The authors declare that there is no conflict of interest.

## Authors’ contributions

BT prepared the proposal, contributed in the data collection, did the statistical analysis and wrote the final manuscript. ET edited the proposal, contributed for data collection, and edited the final manuscript. Both authors read and approved the final manuscript.

## References

[B1] Alzamora-Castro V Battilana G Abugattas R Sialer S Patent ductus arteriosus and high altitude [Abstract] Am J Cardiol 1960 5 761 763 10.1016/0002-9149(60)90052-7 13793062

[B2] Jacek B Jan G Carlos Z Antonio GM Ramon BC Ramon FA Antonio SS Hernan DL Ramiro MD Jaqueline K Patent ductus arteriosus at low and high altitudes: anatomical and haemodynamic features and their implications for transcatheter closure Kardiol Pol 2011 69 5 431 436 21594824

[B3] Daniel B Robert MK, Richard EB, Hal BJ, Bonita FS Congenital heart disease: patent ductus arteriosus Nelson text book of pediatrics 2008 18 Philadelphia: Saunders Elsvier 953 4

[B4] Omar MG Mohamed A Arif H Amjad K Jameel AA Ahmed J Left ventricular dysfunction after closure of large patent Ductus Arteriosus Asian Cardiovasc Thorac Ann 2005 13 24 29 10.1177/021849230501300106 15793046

[B5] Anneli E Eero J Talvikki B Jaana P The influence of percutaneous closure of patent ductus arteriosus on left ventricular size and function: a prospective study using Two- and three-dimensional echocardiography and measurements of serum natriuretic peptides J Am Coll Cardiol 2006 47 1060 6 10.1016/j.jacc.2005.09.067 16516094

[B6] Masura J Walsh KP Thanopoulous B Chan C Bass J Goussous Y Gavora P Hijazi ZM Catheter closure of moderate- to large-sized patent ductus arteriosus using the New amplatzer duct occluder: immediate and short-term results J Am Coll Cardiol 1998 31 4 878 82 10.1016/S0735-1097(98)00013-8 9525563

[B7] Gupta SK Krishnamoorthy KM Tharakan JA Sivasankaran S Sanjay G Bijulal S Anees T Percutaneous closure of patent ductus arteriosus in children: Immediate and short-term changes in left ventricular systolic and diastolic function Ann Pediatr Card 2011 4 2 139 144 10.4103/0974-2069.84652 PMC3180971 21976873

[B8] Takahashi Y Harada K Ishida A Tamura M Tanaka T Takada G Changes in left ventricular volume and systolic function before and after the closure of ductus arteriosus in full-term infants Early Hum Dev 1996 44 1 77 85 10.1016/0378-3782(95)01695-3 8821898

[B9] Myung KP Special tools in evaluation of cardiac patients (Non-invasive techniques): echocardiography Pediatric cardiology for practitioners 2005 5 Philadelphia, PA: Mosby Elsevier 200

[B10] Masura J Tittel P Gavora P Podnar T Long-term outcome of transcatheter patent ductus arteriosus closure using Amplatzer duct occluders Am Heart J 2006 151 3 755.e7 755.e10 10.1016/j.ahj.2005.12.010 16504649

[B11] Patrick JM Lilian S Sandesh PS Derek S Arvind S Patent ductus arteriosus ligation is associated with impaired left ventricular systolic performance in premature infants weighing less than 1000 g J Thorac Cardiovasc Surg 2010 140 1 150 157 10.1016/j.jtcvs.2010.01.011 20363478

[B12] Kim YH Choi HJ Cho Y Lee SB Hyun MC Transient left ventricular dysfunction after percutaneous patent ductus arteriosus closure in children Korean Circ J 2008 38 596 600 10.4070/kcj.2008.38.11.596

[B13] Shahab N Philippe F Istvan S Pierre W Changes in myocardial function and hemodynamics after ligation of the ductus arteriosus in preterm infants J Pediatr 2007 150 597 602 10.1016/j.jpeds.2007.01.035 17517241

[B14] Raghda G Mona A Mohamed H Osama A Ebraheem K Strain rate imaging for the evaluation of left ventricular function after patent ductus arteriosus closure Heart Mirror Journal 2011 5 3 387 392

[B15] Jeong YH Yun TJ Song JM Park JJ Seo DM Koh JK Lee SW Kim MJ Kang DH Song JK Left ventricular remodeling and change of systolic function after closure of patent ductus arteriosus in adults: device and surgical closure Am Heart J 2007 154 3 436 440 10.1016/j.ahj.2007.04.045 17719286

